# Feasibility and diagnostic utility of echocardiographic distal abdominal aortic diastolic flow reversal for assessing aortic regurgitation

**DOI:** 10.1093/ehjimp/qyag176

**Published:** 2026-10-12

**Authors:** Wataru Suzuki, Sakiko Kojima, Reina Takeda, Hirotsugu Mihara, Atomu Tajima, Masahiro Shimoda, Hirofumi Ohashi, Ayako Kunimura, Kentaro Mukai, Yusuke Nakano, Hirohiko Ando, Tetsuya Amano

**Affiliations:** Department of Cardiology, Aichi Medical University, 1-1, Yazakokarimata, Nagakute 480-1195, Japan; Department of Clinical Laboratory, Aichi Medical University, Nagakute, Japan; Department of Clinical Laboratory, Aichi Medical University, Nagakute, Japan; Yokkaichi Heart Clinic, Yokkaichi, Japan; Department of Cardiology, Aichi Medical University, 1-1, Yazakokarimata, Nagakute 480-1195, Japan; Department of Cardiology, Aichi Medical University, 1-1, Yazakokarimata, Nagakute 480-1195, Japan; Department of Cardiology, Aichi Medical University, 1-1, Yazakokarimata, Nagakute 480-1195, Japan; NCD Epidemiology Research Center, Shiga University of Medical Science, Otsu, Japan; Department of Cardiology, Aichi Medical University, 1-1, Yazakokarimata, Nagakute 480-1195, Japan; Department of Cardiology, Aichi Medical University, 1-1, Yazakokarimata, Nagakute 480-1195, Japan; Department of Cardiology, Aichi Medical University, 1-1, Yazakokarimata, Nagakute 480-1195, Japan; Department of Cardiology, Aichi Medical University, 1-1, Yazakokarimata, Nagakute 480-1195, Japan

**Keywords:** abdominal aorta, aortic regurgitation, cardiac magnetic resonance, diastolic flow reversal, echocardiography, holo-diastolic flow reversal

## Abstract

**Aims:**

Although abdominal aortic holo-diastolic flow reversal (HDFR) is a useful marker for aortic regurgitation (AR) severity, guidelines do not specify the optimal measurement site. We compared the feasibility of proximal vs. distal abdominal aortic diastolic flow reversal (DFR) measurements, evaluated the diagnostic accuracy of distal HDFR for severe AR, and assessed its consistency with cardiac magnetic resonance (CMR).

**Methods and results:**

We evaluated 103 patients with moderate (*n* = 81) or severe (*n* = 22) chronic AR. DFR feasibility was significantly higher in the distal than in the proximal segment (94% vs. 54%, *P* < 0.01). In the overall clinical diagnostic yield analysis (*n* = 103), distal DFR demonstrated a significantly higher diagnostic yield for AR severity grading compared with proximal DFR (82% vs. 50%, *P* < 0.01). In an exploratory paired analysis of patients with feasible DFR at both sites (*n* = 56), distal and proximal HDFR demonstrated no statistically significant differences in diagnostic performance for severe AR: sensitivity (92% vs. 85%, *P* = 1.00), specificity (86% vs. 93%, *P* = 0.38), and accuracy (88% vs. 91%, *P* = 0.73). Inter- and intra-observer reproducibility was excellent. Furthermore, echo-derived distal HDFR showed descriptive consistency with CMR-derived HDFR in a small exploratory subgroup (*n* = 12; 92% agreement, κ = 0.82).

**Conclusion:**

Distal abdominal aortic DFR is a simple and highly feasible method for assessing AR severity, potentially overcoming the limitations of proximal DFR assessment in routine clinical practice.

## Graphical Abstract

**Graphical Abstract:**
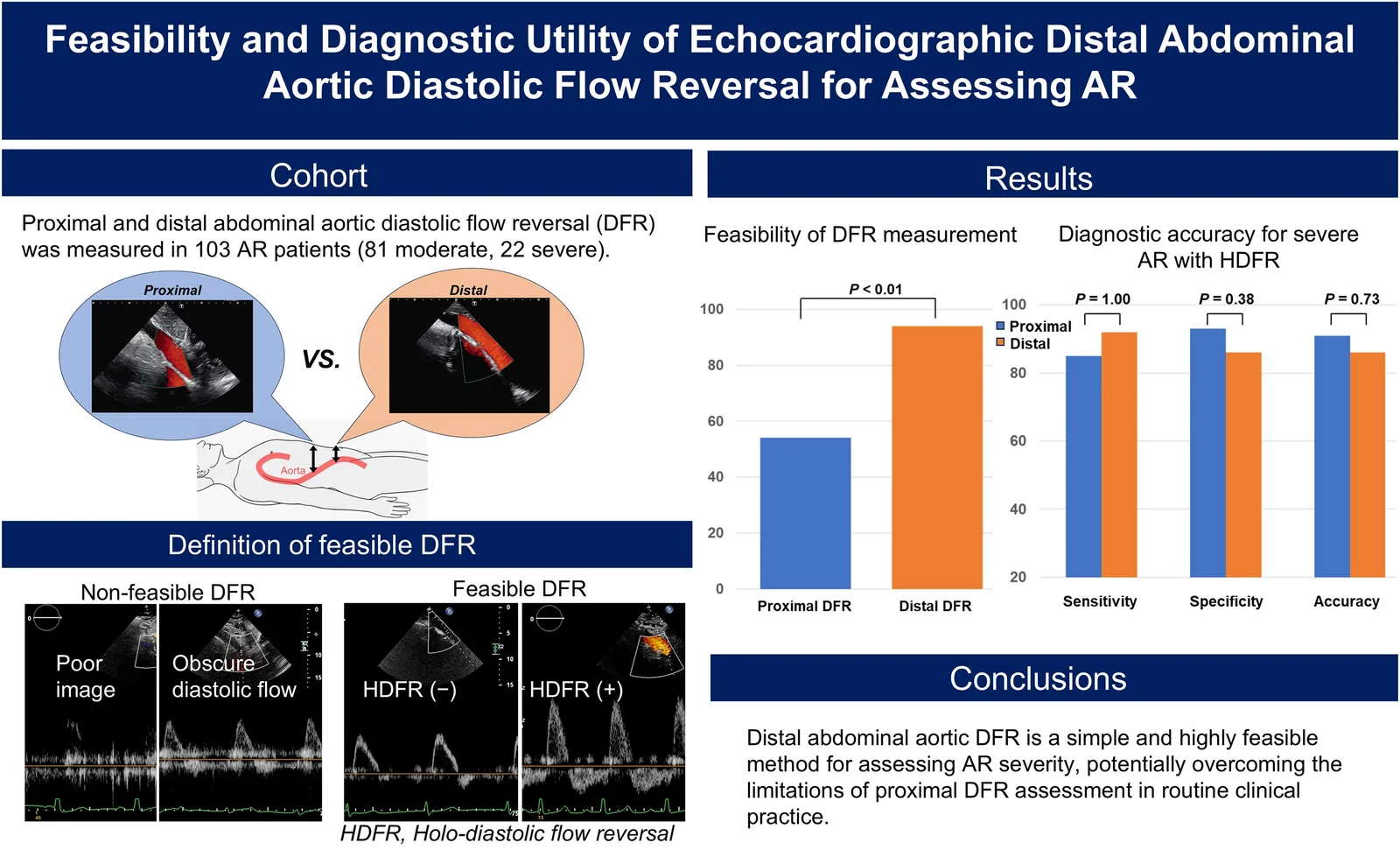

## Introduction

Because the quantitative assessment of aortic regurgitation (AR) by echocardiography—including effective regurgitant orifice area, regurgitant volume, and regurgitant fraction—is often limited by technical challenges and inherent complexity, supplementary diagnostic support using simpler qualitative or semi-quantitative parameters is highly beneficial and integrated into current guidelines and documents.^1,2^ Among these, holo-diastolic flow reversal (HDFR) detected via aortic diastolic flow reversal (DFR) waveform analysis serves as a simple and widely used qualitative indicator.^1–6^ Meanwhile, although the abdominal aorta can be broadly visualized from the proximal to the distal site using echocardiography,^3^ current guidelines and documents do not specify the optimal site for AR evaluation.^1,2^ While previous studies have reported the utility of the proximal abdominal aorta for assessing AR,^7,8^ we hypothesized that the distal abdominal aorta provides easier visualization and evaluation of DFR, given its anatomical and hemodynamic characteristics.

The purpose of this study was to evaluate the clinical utility of distal abdominal aortic DFR compared with the conventional proximal site for routine assessment of AR severity by comparing three key factors: (i) the feasibility of DFR measurement, (ii) diagnostic performance for severe AR, and (iii) agreement with cardiac magnetic resonance (CMR)-derived HDFR—an established, objective marker for severe AR.

## Methods

### Study design

This study was designed as a single-centre, cross-sectional investigation. Consecutive patients who underwent dedicated echocardiographic examinations for the evaluation of chronic AR at Aichi Medical University Hospital between April 2024 and May 2026 were systematically evaluated. In addition to measuring standard AR evaluation parameters in accordance with current guidelines, DFR was measured at both the proximal and distal abdominal aorta. The exclusion criteria were concomitant severe valvular disease other than AR, post-aortic valve surgery, bradycardia, or tachycardia (heart rate ˂40 bpm or >100 bpm), and mild AR. Patients with moderate or severe AR were included in the final analysis to compare the feasibility of DFR measurement between the proximal and distal sites. Furthermore, among patients in whom DFR was measurable at both sites, the diagnostic accuracy for severe AR using HDFR was compared between the proximal and distal abdominal aorta.

As a sub-study, the concordance rate between echo-derived distal HDFR and CMR-derived HDFR was evaluated in a subgroup of the final cohort for whom CMR assessment of AR severity was clinically indicated.

We extracted information on patient background, blood test, and CMR results from the medical records. This study adhered to the principles of the Declaration of Helsinki and was approved by the Ethics Committee of Aichi Medical University (Nagakute, Japan).

### Echocardiographic assessment of AR severity

Echocardiographic examinations were performed by one of the several echocardiographers to acquire image data. Based on these collected data, an echocardiographic cardiologist (W.S.), who was blinded to DFR feasibility and HDFR status, assessed AR severity using an integrative, multiparametric approach in accordance with the American Society of Echocardiography guidelines without considering HDFR.^1^ For concentric jets, quantitative parameters—specifically effective regurgitant orifice area (EROA) and regurgitant volume (RV)—were prioritized to distinguish moderate from severe AR. In cases with eccentric jets or technical limitations where quantitative measurements were unreliable, adjudication relied on semi-quantitative metrics (vena contracta width and pressure half-time) integrated with left ventricular size. To assess intra-observer reliability, W.S. re-evaluated a randomly selected subset of 30 patients at least 1 month after the initial analysis in a blinded manner. To assess inter-observer reliability for AR severity classification, a second cardiologist (K.M.) independently evaluated a randomly selected subset of 30 patients using the same standardized criteria, while remaining blinded to the clinical and HDFR data. To evaluate agreement across the entire spectrum of AR severity, this subset was selected from the initial screening cohort prior to applying the exclusion criteria (thereby including patients with mild, moderate, and severe AR). Separately, these DFR parameters were evaluated by the examining sonographers. For all echocardiographic evaluations, either the EPIQ Elite with an X5-1 probe (Philips Ultrasound, Bothell, WA, USA) or the Aplio i900 with a 6S1 probe (Canon Medical Systems, Otawara, Japan) was utilized.

### Proximal and distal DFR

Following previous reports, the proximal abdominal aorta was defined as the segment near the celiac artery visualized from the subcostal approach, whereas the distal abdominal aorta was defined as the segment proximal to the common iliac artery bifurcation visualized from the periumbilical approach^3,9^ (*Figure 1*). DFR was measured using the same echocardiographic equipment and probes as those used for the severity assessment of AR with the patient in the supine position. Measurements were initiated with a sample volume of 3.0–4.0 mm and the minimum wall filter setting; if excessive noise was present, the filter threshold was increased incrementally. The abdominal aorta was visualized under colour Doppler guidance, and blood flow velocity waveforms were obtained using pulsed-wave Doppler in a view co-axial with the abdominal aorta. The feasibility of DFR measurement was classified as follows: measurements were defined as non-feasible in cases of: ‘poor image’, where systolic anterograde abdominal aortic flow could not be visualized even after decreasing the colour Doppler velocity scale to 30–50 cm/s, and a corresponding systolic anterograde flow waveform could not be displayed by pulsed-wave Doppler; or ‘obscure diastolic flow’, where adequate systolic anterograde flow was visualized by colour Doppler and displayed by pulsed-wave Doppler, but the velocity waveform showed simultaneous coexistence of both anterograde and retrograde signals during diastole (*Figure 2*). Conversely, cases with distinct blood flow waveforms obtained during both systole and diastole were defined as feasible. Among the feasible cases, the presence of a holo-diastolic reversal flow waveform continuing until end-diastole was defined as HDFR-positive when observed for at least three consecutive cardiac cycles (*Figure 2*). In accordance with standard guidelines, abdominal aortic HDFR was evaluated as a qualitative binary parameter (present vs. absent) based on temporal flow persistence throughout diastole.^1,2^

**Figure 1:**
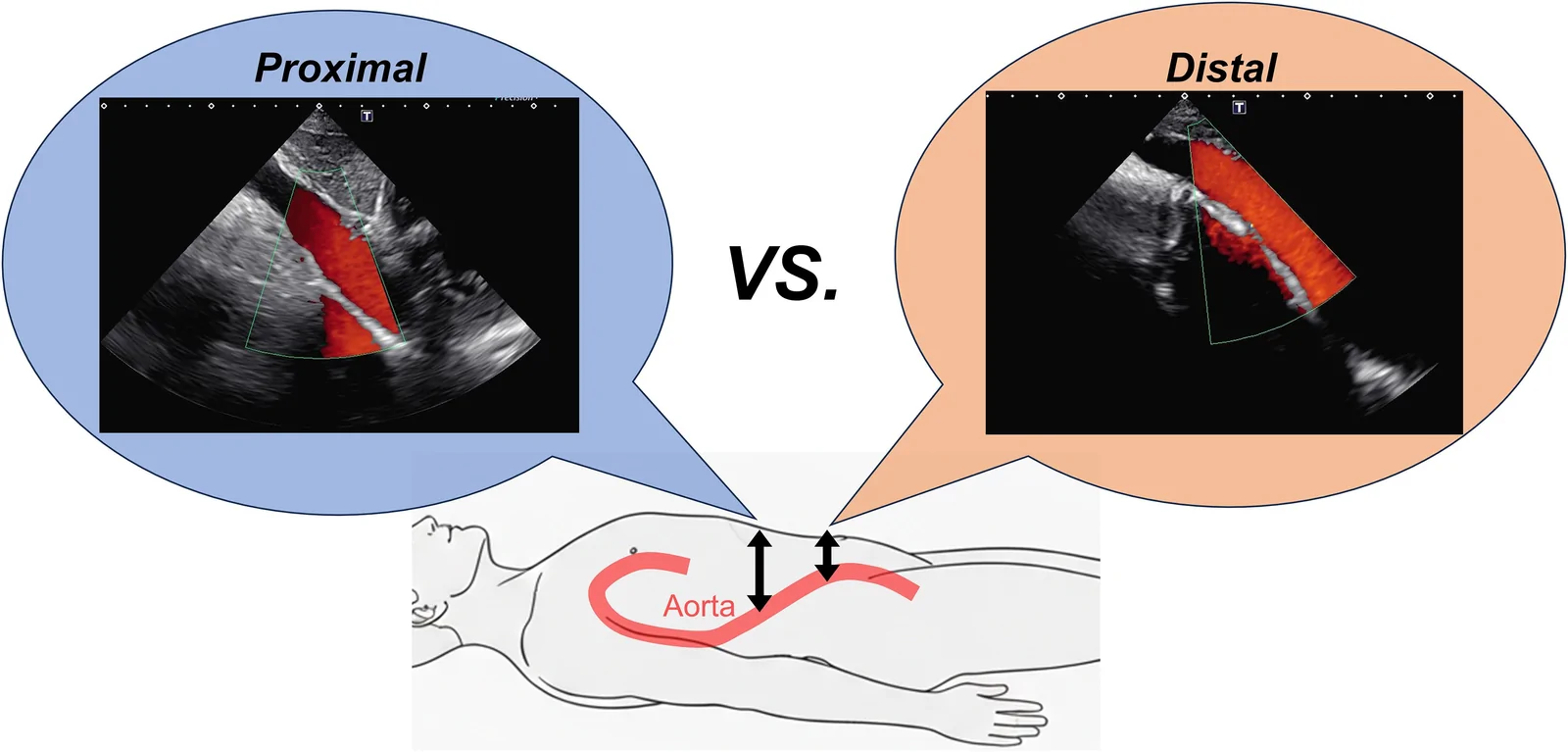
Ultrasonographic images of proximal and distal abdominal aorta. The proximal and distal abdominal aorta were defined as the segments near the celiac artery (visualized via the subcostal approach) and proximal to the common iliac artery bifurcation (visualized via the periumbilical approach), respectively.

**Figure 2:**
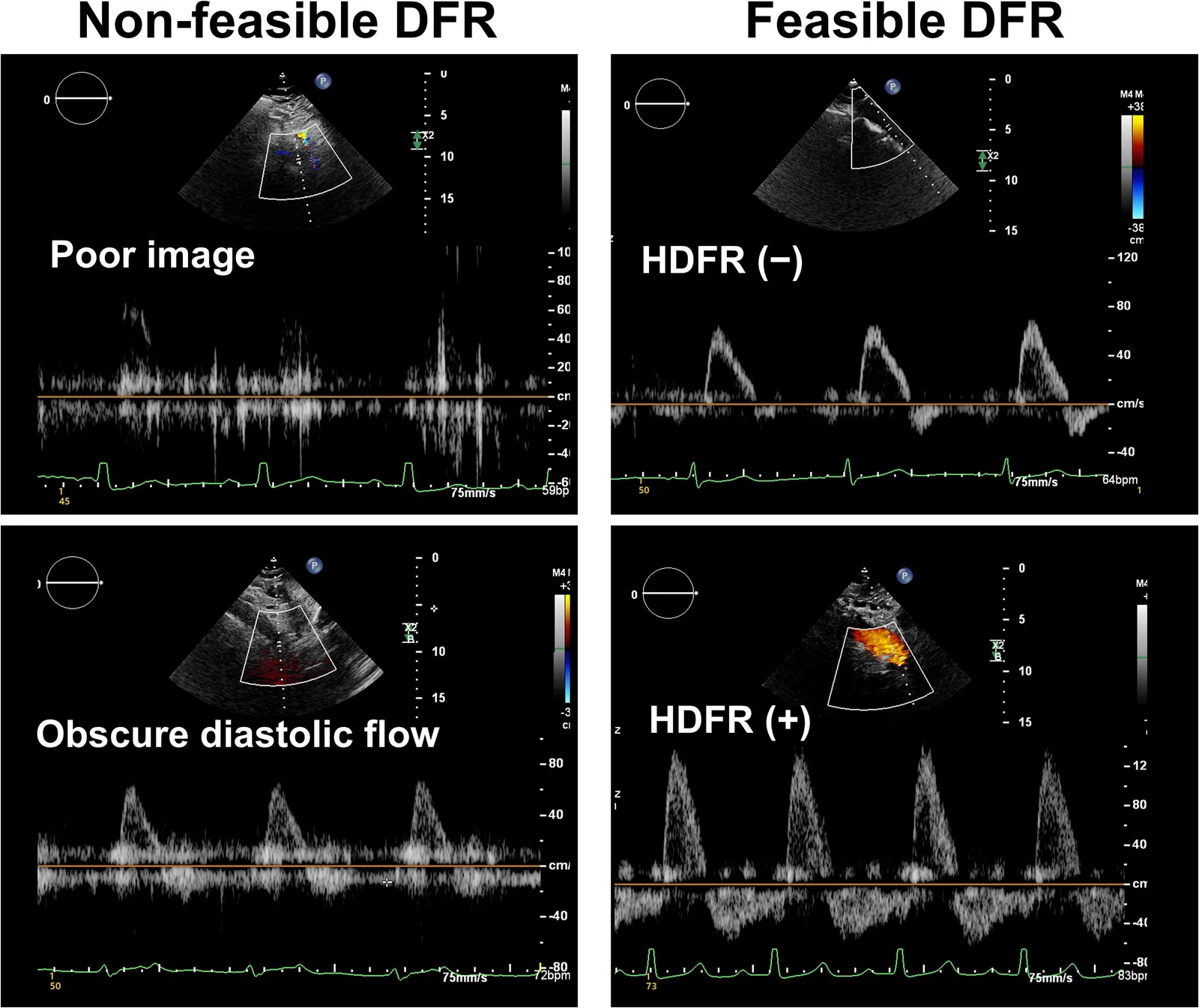
Representative cases of feasible and non-feasible DFR measurements. DFR measurement feasibility was classified as feasible (distinct systolic and diastolic waveforms) or non-feasible. Non-feasibility was defined as ‘poor image’ (systolic anterograde flow could not be displayed by colour or pulsed-wave Doppler, even at a velocity scale of 30–50 cm/s) or ‘obscure diastolic flow’ (adequate systolic flow was present, but diastolic waveforms showed simultaneous anterograde and retrograde signals). Among feasible cases, HDFR-positive was defined as the presence of a holo-diastolic reversal flow waveform continuing until end-diastole.

### Reproducibility of DFR measurement

To evaluate inter-observer variability, 20 randomly selected patients were assessed. For each patient, independent observers measured the proximal and distal DFR to determine the feasibility of DFR measurement and the presence or absence of HDFR. The assessment results of the observers were then compared. To evaluate intra-observer variability, the same echocardiographer (S.K.) performed a re-interpretation of the stored DFR waveform images from his/her initial examinations after an interval of at least 1 month. The feasibility of proximal and distal DFR measurements, as well as the presence or absence of HDFR, were re-determined and compared with the initial results. Inter- and intra-observer agreements for the feasibility of DFR measurement and the presence of HDFR were quantified using Cohen’s κ coefficient.

### CMR assessment of HDFR

Among the enrolled patients, those in whom CMR was deemed clinically necessary at the discretion of the attending physicians were included in this CMR sub-analysis. All CMR studies were performed using a 1.5-T cardiac-dedicated system (Avanto, Siemens Medical Solutions, Erlangen, Germany) according to standard protocols.^10^ The phase-contrast velocity-encoding sequence was prescribed in a plane perpendicular to the descending aorta at the level of the main pulmonary artery according to the standard protocols and previous studies.^10–12^ Flow time curves were used to identify presence or absence of HDFR in a separate orthogonal slice (see Supplementary data online, *Figure S1*). Breath hold sequences were used. All CMR analyses were performed offline, using dedicated software. Contours were drawn manually in each patient. To avoid bias, investigators were blinded to the TTE results. Following standard protocols and previous studies, severe AR based on CMR was defined by the presence of HDFR.^10–12^

### Statistical analysis

Continuous variables were expressed as medians with 25th and 75th percentiles (inter-quartile range), and categorical variables were presented as frequencies and percentages. Continuous variables were compared using the Mann–Whitney *U* test, and categorical variables were compared using the χ^2^ test or Fisher’s exact test, as appropriate. For paired categorical observations between proximal and distal DFR measurement sites within the same patient—including measurement feasibility, overall clinical diagnostic yield (intention-to-diagnose analysis categorizing non-feasible measurements as diagnostic failures), HDFR detection rates, and diagnostic performance parameters (sensitivity, specificity, and accuracy)—McNemar’s test was performed. Inter- and intra- observer reliability for AR severity classification was assessed using simple agreement rates and weighted κ coefficients. Inter- and intra-observer variabilities for DFR parameters, as well as the comparison of HDFR between echocardiography and CMR, were assessed using simple agreement rates and Cohen’s κ coefficients. Ninety-five percent confidence intervals (95% CIs) were calculated where appropriate. All statistical analyses were performed using SPSS software (version 28.0.1.1; IBM Corp., Armonk, NY, USA). A *P* value of <0.05 was considered statistically signiﬁcant.

## Results

### Baseline characteristics and AR severity

A total of 178 consecutive patients with AR were screened (*Figure 3*). After excluding 2 patients with concomitant severe aortic stenosis and 73 patients with mild AR (of whom 16 were included in preliminary reproducibility testing), a total of 103 patients (81 with moderate AR and 22 with severe AR) constituted the primary study cohort. The overall median age was 76 years, and 34% were women. Clinical comorbidities (predominantly hypertension) were similarly distributed between the moderate and severe AR groups (*Table 1*). Distal abdominal aortic DFR assessment was feasible in 97 patients (94%), whereas proximal assessment was feasible in 56 patients (54%), yielding a dual-site feasibility subgroup of 56 patients for paired comparisons (*Figure 3*). Compared with the moderate AR group, the severe AR group demonstrated significantly higher systolic blood pressure [142 (132–157) mmHg vs. 128 (114–139) mmHg, *P* < 0.01] and pulse pressure [83 (65–96) mmHg vs. 58 (50–66) mmHg, *P* < 0.01], but significantly lower diastolic blood pressure [61 (52–66) mmHg vs. 66 (61–77) mmHg, *P* = 0.02] (*Table 1*). Regarding echocardiographic findings, leaflet degeneration was the predominant AR aetiology (70%), with similar distribution across groups. The severe AR group had significantly larger left ventricular end-diastolic and end-systolic volumes than the moderate AR group [196 (126–225) mL vs. 104 (88–123) mL, *P* < 0.01 and 93 (69–118) mL vs. 44 (33–60) mL, *P* < 0.01, respectively], whereas left ventricular ejection fraction was significantly lower in the severe AR group [53% (44–60%) vs. 59% (54–64%), *P* = 0.01] (*Table 2*).

**Figure 3:**
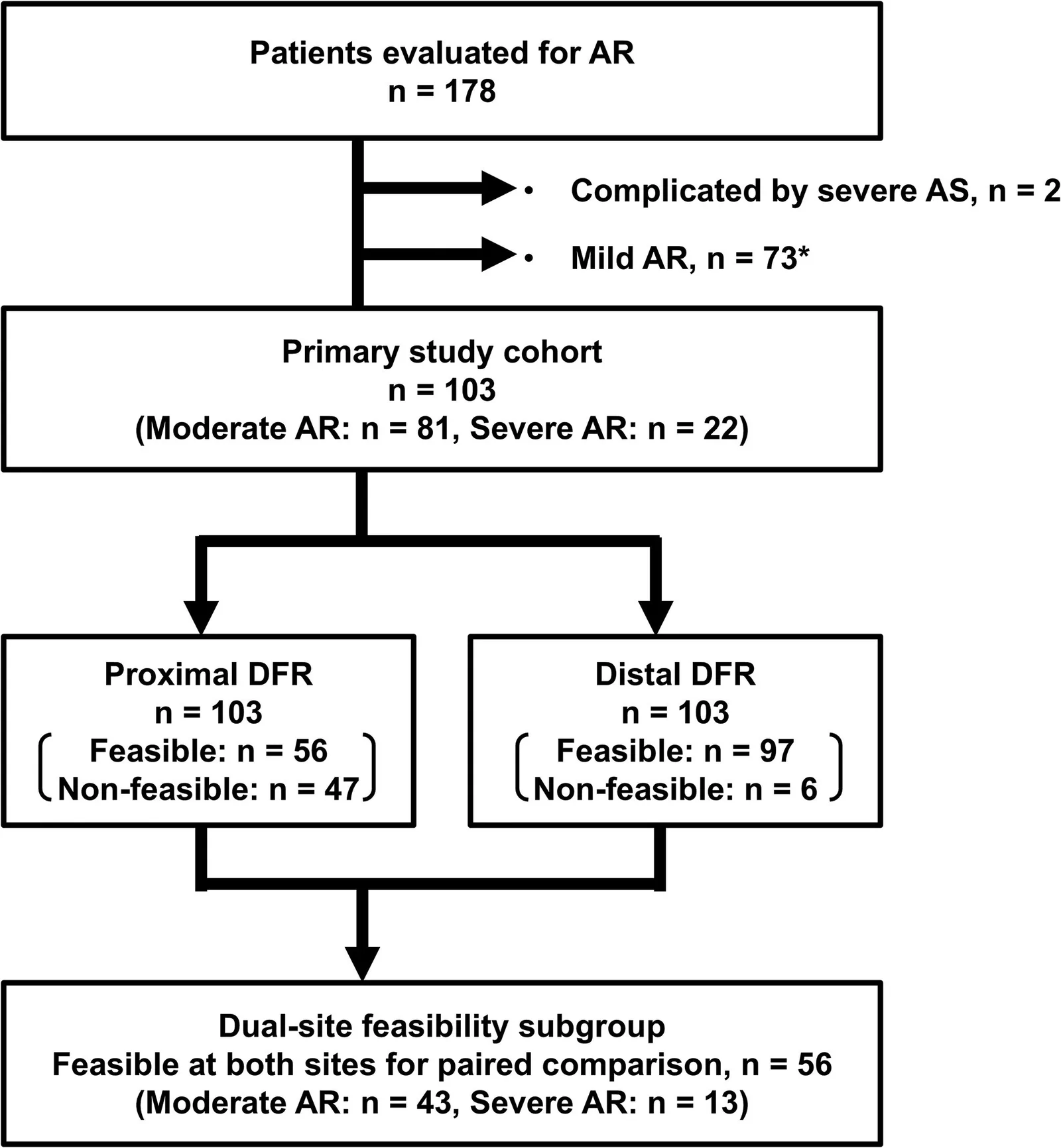
Study flowchart. Flowchart detailing the selection of 103 patients with moderate (*n* = 81) or severe (*n* = 22) chronic AR from 178 screened individuals. The feasibility of proximal vs. distal abdominal aortic DFR assessment and the breakdown for the paired dual-site comparison subgroup (*n* = 56) are shown. *Sixteen of the 73 patients with mild AR were included in preliminary reproducibility testing for echocardiographic AR grading prior to primary study cohort selection.

**Table 1:** Patients baseline characteristics

Variable	All patients (*n* = 103)	Moderate AR (*n* = 81)	Severe AR (*n* = 22)	*P* value
Age, years	76 (65–83)	76 (68–84)	71 (59–77)	0.02
Woman, *n* (%)	34 (33)	28 (35)	6 (27)	0.52
Body surface area, m^2^	1.61 (1.44–1.73)	1.61 (1.43–1.73)	1.60 (1.48–1.73)	0.58
Hypertension, *n* (%)	74 (72)	55 (68)	19 (86)	0.09
Dyslipidaemia, *n* (%)	31 (30)	26 (32)	5 (23)	0.40
Diabetes, *n* (%)	13 (13)	12 (15)	1 (5)	0.29
Prior myocardial infarction, *n* (%)	6 (6)	6 (7)	0 (0)	0.34
Systolic blood pressure, mmHg	132 (115–146)	128 (114–139)	142 (132–157)	< 0.01
Diastolic blood pressure, mmHg	65 (60–76)	66 (61–77)	61 (52–66)	0.02
Pulse pressure, mmHg	61 (52–74)	58 (50–66)	83 (65–96)	< 0.01
Heart rate, bpm	71 (65–77)	70 (63–78)	72 (69–77)	0.25
Haemoglobin, g/dL	13.0 (11.1–14.0)	12.9 (11.0–14.1)	13.1 (11.2–13.9)	0.78
eGFR, mL/min/1.73 m^2^	59 (42–75)	57 (39–75)	64 (49–74)	0.43

AR, aortic regurgitation; bpm, beats per minute; eGFR, estimated glomerular filtration rate.

**Table 2:** Comparison of echocardiographic data between moderate and severe AR

Variable	All patients (*n* = 103)	Moderate AR (*n* = 81)	Severe AR (*n* = 22)	*P* value
Left ventricle				
LVEDD, mm	49 (44–55)	48 (43–53)	60 (51–63)	<0.01
LVESD, mm	34 (28–40)	33 (28–38)	46 (35–48)	<0.01
IVSD, mm	11 (10–12)	11 (10–12)	12 (10–12)	0.11
PWD, mm	11 (10–12)	11 (10–12)	12 (10–12)	0.09
LVEDV, mL	113 (94–141)	104 (88–123)	196 (126–225)	<0.01
LVESV, mL	50 (34–71)	44 (33–60)	93 (69–118)	<0.01
LVEF, %	58 (51–64)	59 (54–64)	53 (44–60)	0.01
Diastolic function				
LAVI, mL/m^2^	32 (26–41)	31 (26–43)	33 (27–36)	0.81
*E*/*e*´	10 (8–13)	10 (8–13)	10 (8–13)	0.58
Aetiology, *n* (%)				
Leaflet degeneration^a^	72 (70)	56 (69)	16 (72)	0.75
Aortic root dilation	13 (13)	9 (11)	4 (18)	0.47
Bicuspid	4 (4)	4 (5)	0 (0)	0.23
Others/unclear	14 (14)	12 (15)	2 (9)	0.49
Aortic regurgitation				
Vena contracta, mm	5.0 (4.3–5.8)	4.7 (4.1–5.3)	6.3 (5.8–7.4)	< 0.01
Pressure half-time, s	493 (402–605)	542 (441–659)	402 (264–449)	< 0.01
EROA, cm^2^	0.17 (0.13–0.24)	0.15 (0.13–0.20)	0.36 (0.27–0.46)	< 0.01
RV, mL	36 (30–48)	33 (29–41)	63 (50–77)	< 0.01
RF, %	45 (35–52)	42 (34–47)	59 (56–64)	< 0.01

^a^Leaflet degeneration includes focal prolapse, calcification, and restricted leaflet motion.

AR, aortic regurgitation; EROA, effective regurgitant orifice area; IVSD, interventricular septal diameter; LAVI, left atrial volume index; LVEDD, left ventricular end-diastolic diameter; LVEDV, left ventricular end-diastolic volume; LVEF, left ventricular ejection fraction; LVESD, left ventricular end-systolic diameter; LVESV, left ventricular end-systolic volume; PWD, posterior wall diameter; RF, regurgitant fraction; RV, regurgitant volume.

For the inter- and intra-observer reliability assessment of AR severity classification, in a randomly selected subset of 30 patients, W.S. categorized 16 (53%) as mild, 9 (30%) as moderate, and 5 (17%) as severe. The inter-observer agreement between W.S. and K.M. demonstrated excellent reliability, with a weighted κ coefficient of 0.92 (95% CI: 0.82 to 1.00, *P* < 0.01) and a simple agreement rate of 90%. Furthermore, the intra-observer agreement (W.S.) demonstrated excellent reliability, with a weighted κ coefficient of 0.91 (95% CI: 0.81 to 1.00, *P* < 0.01) and a simple agreement rate of 93%.

### Feasibility of DFR measurement and diagnostic accuracy for severe AR with HDFR

The feasibility of DFR measurement was significantly higher in the distal site than in the proximal site (54% in the proximal vs. 94% in the distal, *P* < 0.01) (*Table 3* and *Figure 4*). Among the 47 cases where proximal DFR was non-feasible, the underlying causes were a poor image in 20 cases (19%) and an obscure diastolic flow in 27 cases (26%). Notably, of these 47 cases where proximal DFR was non-feasible, 41 (87%) were feasible for DFR measurement at the distal site. Conversely, among the 6 cases in whom distal DFR was non-feasible, the underlying causes were poor image quality in 1 case (1%) and obscure diastolic flow in 5 cases (5%). In the overall clinical diagnostic yield analysis across the entire cohort (*n* = 103), incorporating both feasibility and correct diagnostic classification, distal DFR demonstrated a significantly higher diagnostic yield for AR severity grading compared with proximal DFR (50% in the proximal vs. 82% in the distal, *P* < 0.01). Among the 97 patients with feasible distal DFR (76 with moderate AR and 21 with severe AR), HDFR was positive in 32 patients, including 12 with moderate AR and 20 with severe AR. The diagnostic performance of distal HDFR for severe AR demonstrated a sensitivity of 95%, specificity of 84%, and accuracy of 87%. Furthermore, patients presenting with distal HDFR exhibited significantly greater quantitative AR severity metrics than those without distal HDFR [EROA: 0.25 (0.24–0.34) cm^2^ vs. 0.15 (0.15–0.18) cm^2^, *P* < 0.01; RV: 49 (46–60) mL vs. 33 (32–37) mL, *P* < 0.01].

**Figure 4:**
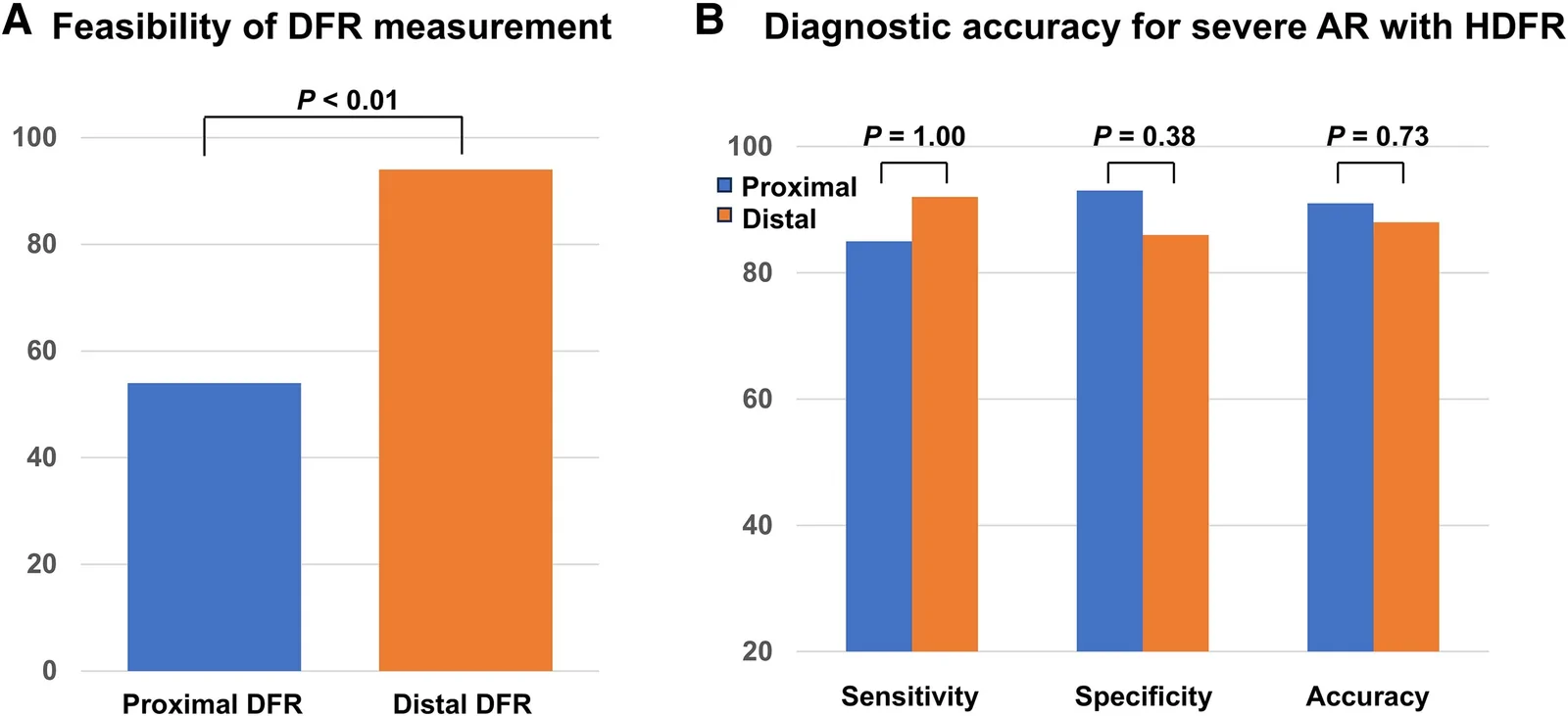
Feasibility of DFR measurement and diagnostic accuracy of HDFR for severe AR at proximal vs. distal abdominal aorta. (*A*) The feasibility of DFR measurement was significantly higher in the distal site than in the proximal site (54% in the proximal vs. 94% in the distal, *P* < 0.01). (*B*) In patients with feasible proximal and distal DFR, proximal and distal HDFR demonstrated no statistically significant differences in diagnostic parameters for severe AR, with a sensitivity of 85% vs. 92% (*P* = 1.00), a specificity of 93% vs. 86% (*P* = 0.38), and an accuracy of 91% vs. 88% (*P* = 0.73), respectively.

**Table 3:** Feasibility of DFR measurement and diagnostic accuracy of HDFR for severe AR at proximal vs. distal abdominal aorta

Variable	Proximal	Distal	*P* value
Feasibility of DFR measurement (*n* = 103), % (*n*/*N*)	54% (56/103)	94% (97/103)	< 0.01
Clinical diagnostic yield for AR severity grading (*n* = 103)^a^, % (*n*/*N*)	50% (51/103)	82% (84/103)	< 0.01
Frequency of HDFR in moderate and severe AR among cases with feasible DFR in both sites			
Moderate AR (*n* = 43), % (*n*/*N*)	7% (3/43)	14% (6/43)	0.38
Severe AR (*n* = 13), % (*n*/*N*)	85% (11/13)	92% (12/13)	1.00
Diagnostic accuracy for severe AR with HDFR among cases with feasible DFR in both sites (*n* = 56)			
Sensitivity (95% CI)	85% (55–98%)	92% (62–100%)	1.00
Specificity (95% CI)	93% (81–99%)	86% (72–95%)	0.38
Accuracy (95% CI)	91% (80–96%)	88% (76–95%)	0.73

^a^Clinical diagnostic yield was defined as the proportion of patients in whom DFR measurement was both technically feasible and correctly classified regarding AR severity (severe vs. moderate AR) across the entire cohort.

AR, aortic regurgitation; CI, confidence interval; DFR, diastolic flow reversal; HDFR, holo-diastolic flow reversal. 95% confidence intervals were calculated using the Clopper–Pearson exact method.

In patients where DFR was feasible in both the proximal and distal sites (43 with moderate AR and 13 with severe AR), the detection rate of HDFR in moderate AR showed no significant difference between the proximal and distal sites (7% vs. 14%, *P* = 0.38). Similarly, the detection rate of HDFR in severe AR showed no significant difference between the two sites (85% vs. 92%, *P* = 1.00) (*Table 3*). Furthermore, in an exploratory analysis of patients with feasible proximal and distal DFR, proximal and distal HDFR demonstrated no statistically significant differences in diagnostic parameters for severe AR, with a sensitivity of 85% vs. 92% (*P* = 1.00), a specificity of 93% vs. 86% (*P* = 0.38), and an accuracy of 91% vs. 88% (*P* = 0.73), respectively (*Table 3* and *Figure 4*).

### Inter- and intra-observer reproducibility of HDFR

Inter-observer variability for the feasibility of DFR measurement was minimal for both proximal and distal measurements, with percentage agreements of 85% (17 of 20) and 90% (18 of 20), respectively. The agreement for proximal DFR feasibility was substantial (κ = 0.70, 95% CI: 0.37 to 1.00). In contrast, the κ value for distal DFR feasibility was low (κ = −0.05) despite the high percentage agreement, owing to an imbalanced category distribution (prevalence paradox). Among cases where distal DFR was considered feasible by both observers, agreement regarding the presence of HDFR was excellent, with a percentage agreement of 94% (17 of 18) and a κ coefficient of 0.89 (95% CI: 0.57 to 1.00) (*Table 4*).

**Table 4:** Inter-observer and intra-observer variability of DFR feasibility and HDFR agreement, and comparison with CMR

	Percentage of agreement, % (*n*/*N*)	Cohen’s κ coefficient (95% CI)
Inter-observer variability		
Feasibility of proximal DFR	85% (17/20)	0.70 (0.37 to 1.00)
Feasibility of distal DFR	90% (18/20)	−0.05 (−0.14 to 0.00)
HDFR agreement among cases with feasible distal DFR by both observers	94% (17/18)	0.89 (0.57 to 1.00)
Intra-observer variability		
Feasibility of proximal DFR	90% (18/20)	0.80 (0.49 to 1.00)
Feasibility of distal DFR	100% (20/20)	-^a^
HDFR agreement among cases with feasible distal DFR in both observations	95% (18/19)	0.89 (0.66 to 1.00)
Comparison with CMR		
Distal HDFR vs. CMR-HDFR	92% (11/12)	0.82 (0.39 to 1.00)

CI, confidence interval; CMR, cardiovascular magnetic resonance; DFR, diastolic flow reversal; HDFR, holo-diastolic flow reversal.

^a^Not calculable due to 100% agreement (constant categories in cross-tabulation).

Intra-observer variability analysis based on offline re-interpretation also demonstrated excellent reproducibility of image interpretation. The feasibility assessment of proximal DFR showed a percentage agreement of 90% (18 of 20), representing substantial agreement (κ = 0.80, 95% CI: 0.49 to 1.00). Feasibility assessment of distal DFR achieved complete agreement (100%, 20 of 20). Among cases where distal DFR was feasible in both observations, agreement regarding the presence or absence of HDFR was excellent, with a percentage agreement of 95% (18 of 19) and a κ coefficient of 0.89 (95% CI: 0.66 to 1.00) (*Table 4*).

### Comparison of HDFR between echocardiography and CMR

Of the final cohort, 13 patients underwent CMR for AR evaluation. In one case, distal DFR measurement by echocardiography was non-feasible; thus, the agreement between echo-derived distal HDFR and CMR-derived HDFR was analysed in the remaining 12 patients in whom distal DFR was feasible. Overall, distal HDFR was detected in 7 patients by echocardiography, and HDFR was observed in 8 patients by CMR. In an exploratory analysis, echo-derived distal HDFR demonstrated favourable cross-modality agreement with CMR-derived HDFR regarding the presence or absence of HDFR (92%, 11 of 12), with a κ coefficient of 0.82 (95% CI: 0.39 to 1.00) (*Table 4*).

## Discussion

The present study yielded several important findings regarding the assessment of AR. First, we demonstrated that the measurement of DFR in the distal abdominal aorta is highly feasible and provides a significantly higher overall clinical diagnostic yield compared with the proximal site. Second, in an exploratory analysis of feasible cases, distal HDFR demonstrated no statistically significant differences in diagnostic parameters for severe AR relative to proximal HDFR, while offering substantially superior technical feasibility.

In this study, the rate of poor imaging of the proximal or distal abdominal aortic waveform was 19%. Previous screening studies for abdominal aortic aneurysms using ultrasound in non-fasting subjects reported incomplete visualization rates ranging from 17% to 33%.^13–15^ Although the evaluation targets are not strictly identical, our rate of poor imaging is considered generally reasonable. The high feasibility of distal DFR measurement can be attributed to both anatomical and hemodynamic factors. From an anatomical perspective, the lower abdomen is more compressible and compliant than the epigastric region; this allows for greater flexibility in probe positioning, making it easier to achieve optimal co-axial alignment between the aorta and the ultrasound beam, and thereby facilitating the avoidance of poor image quality. Additionally, the distal abdominal aorta is closer to the skin surface than the proximal abdominal aorta, which enhances ultrasound beam penetration and minimizes artefact interference. Because DFR involves low-velocity blood flow, measuring it closer to the skin surface—where noise is less likely to interfere—is advantageous for avoiding obscure diastolic flow. From a hemodynamic perspective, the impact of blood flow into the abdominal aortic branches (celiac artery, superior mesenteric artery, and inferior mesenteric artery) plays a critical role. The proximal abdominal aorta lies upstream of these major branches. Because these branches supply rich peripheral vascular beds with low resistance, they maintain persistent forward perfusion during diastole. This creates a ‘runoff effect’ that results in detectable diastolic forward flow within the proximal aorta.^3,9,16^ This runoff-induced forward flow can overlap temporally with AR-driven HDFR, making it clinically challenging to differentiate true HDFR from background noise. Conversely, the distal abdominal aorta is unaffected by this branch-related runoff effect, thereby providing a much clearer and well-defined assessment of DFR and thereby avoiding obscure diastolic flow.

### Reproducibility and feasibility of DFR and HDFR measurements

This study demonstrated excellent inter- and intra-observer reproducibility for abdominal aortic DFR and HDFR evaluations, underscoring the clinical robustness and reliability of this method in routine practice. Notably, regarding the inter-observer feasibility of distal DFR, the kappa coefficient was low (κ = −0.05) despite a high overall agreement of 90%. This discrepancy represents the kappa paradox, where a high baseline agreement mathematically suppresses the kappa value by inflating the probability of chance agreement. Therefore, this low kappa value does not indicate poor reliability, but rather reflects the exceptionally high overall feasibility of distal DFR tracking in our cohort. Furthermore, when distal DFR was successfully obtained, inter-observer agreement for the presence of HDFR was remarkably high (κ = 0.89), confirming the diagnostic stability of this index. It should be noted that because intra-observer variability was evaluated using stored Doppler images, it specifically reflects the reproducibility of image interpretation rather than signal re-acquisition.

### Comparison of HDFR between echocardiography and CMR

Another notable finding is the descriptive concordance between echo-derived distal HDFR and CMR-derived HDFR in a small subgroup. CMR-derived HDFR is widely accepted as an objective and highly reproducible parameter for assessing severe AR.^10–12^ Although echocardiographic DFR relies on flow velocity patterns while CMR evaluates flow volume at a different anatomical site—meaning echocardiography does not replace CMR—their observed consistency provides preliminary supportive evidence that distal HDFR reflects the global hemodynamic burden of severe AR. However, given the small sample size and potential selection bias inherent in clinically indicated CMR referrals, these findings should be interpreted as descriptive observations rather than a robust validation.

### Clinical integration of distal HDFR in routine practice

In routine clinical practice, AR severity evaluation relies on an integrative, multiparametric approach, within which distal HDFR should serve as a practical complementary tool rather than a standalone criterion. When regurgitant jet evaluation is technically challenging (e.g. due to poor acoustic windows or eccentric jets) or when proximal DFR assessment is limited by inadequate visualization or obscure diastolic flow, evaluating distal HDFR might provide valuable supplementary diagnostic information.

### Limitations

First, this single-centre study had a small sample size, requiring larger, multicentre validation; in particular, the CMR sub-analysis was performed in a smaller subset based on clinical indication, which may have introduced selection bias. Second, although guidelines recommend using HDFR as an ancillary index for AR severity, we excluded it from our criteria evaluated by a single investigator to avoid circular reasoning in assessing its accuracy. Nonetheless, the guideline-based classification allowed for accurate severity grading without this index, as supported by clinical data (pulse pressure, left ventricular size, and ejection fraction) and excellent inter- and intra- observer reproducibility. Third, pre-examination fasting was not instructed. While bowel gas might have limited the acoustic window for failed distal DFR tracking,^13–15^ routine echocardiography does not require fasting; thus, our protocol may better reflect real-world clinical practice. Fourthly, other potential confounders of HDFR—such as heart rate, shunt diseases, aortic diameter, and arterial stiffness^3,17^—were not fully evaluated. In this relatively elderly population, systemic hemodynamics (including blood pressure, arterial compliance, peripheral resistance, and vascular disease) could potentially influence abdominal aortic flow patterns. The impact of these vascular characteristics on DFR measurement sites warrants future investigation. Finally, classifying DFR feasibility retains some subjectivity in borderline cases. While standardized Doppler settings and observer blinding minimized bias, future studies with automated waveform analysis could offer more objective evaluation.

## Conclusions

DFR in the distal abdominal aorta holds potential as a simple and highly feasible index for assessing AR severity, offering comparable diagnostic utility while overcoming the limitations of proximal DFR evaluation in routine clinical practice. Further validation in larger cohorts using independent reference standards for AR severity is warranted.

## Supplementary Material

qyag176_Supplementary_Data

## Data Availability

Data available on request.

## Lead author biography

Wataru Suzuki, MD, PhD, is a cardiologist and clinician-scientist at Aichi Medical University, Japan. Specialized in echocardiography, his clinical work focuses on structural heart disease (SHD), with a primary research interest in the interplay between valvular heart disease and coronary blood flow dynamics using echocardiographic techniques.
